# Functional Characterization of Parallel Fiber-Purkinje Cell Synapses in Two Friedreich’s Ataxia Mouse Models

**DOI:** 10.1007/s12311-025-01796-0

**Published:** 2025-02-05

**Authors:** Donald J. Joseph, Elizabeth Mercado-Ayon, Liam Flatley, Angela N. Viaene, Juliette Hordeaux, Eric D. Marsh, David R. Lynch

**Affiliations:** 1 Division of Neurology, Department of Pediatrics, The Children’s Hospital of Philadelphia, Philadelphia, PA 19104 USA; 2 Department of Neurology, Perelman School of Medicine, University of Pennsylvania, Philadelphia, PA 19104 USA; 3 Department of Pathology and Laboratory Medicine, Children’s Hospital of Philadelphia, Philadelphia, PA 19104 USA; 4 Department of Pathology and Laboratory Medicine, Perelman School of Medicine, University of Pennsylvania, Philadelphia, PA 19104 USA; 5 Department of Pediatrics and Neurology, Perelman School of Medicine, The Children’s Hospital of Philadelphia, University of Pennsylvania, Philadelphia, PA 19104 USA

**Keywords:** Friedreich’s ataxia, Frataxin, Synaptic transmission, Long-term plasticity, Cerebellum, And Mitochondria

## Abstract

Friedreich ataxia (FRDA) is an autosomal recessive disorder caused by GAA expansions in the *FXN* gene, which codes for the protein frataxin (FXN). These mutations reduce FXN expression, leading to mitochondrial dysfunction and multisystemic disease. Accumulating evidence suggests that neuronal dysfunction, rather than neuronal death, may drive the neurological phenotypes of FRDA, but the mechanisms underlying such neurological phenotypes remain unclear. To investigate the neural circuit basis of this dysfunction, we employed field recordings to measure Purkinje cell (PC) function and synaptic properties along with western blotting and immunohistochemistry to determine their density and structure in two established FRDA mouse models, the shRNA-frataxin (FRDAkd) and the frataxin knock in-knockout (KIKO) mice. Western blotting demonstrated subtle changes in mitochondrial proteins and only a modest reduction in the density of calbindin positive cells PCs in the cerebellar cortex of the FRDAkd mice, with no change in the density of PCs in the KIKO mice. Though PC density differed slightly in the two models, field recordings of parallel fiber-PC synapses in the molecular layer demonstrated concordant hypo-excitability of basal synaptic transmission and impairments of long-term plasticity using induction protocols associated with both potentiation and depression of synaptic strength. These results indicate that synaptic instability might be a common feature in FRDA mouse models.

**Graphical Abstract:**

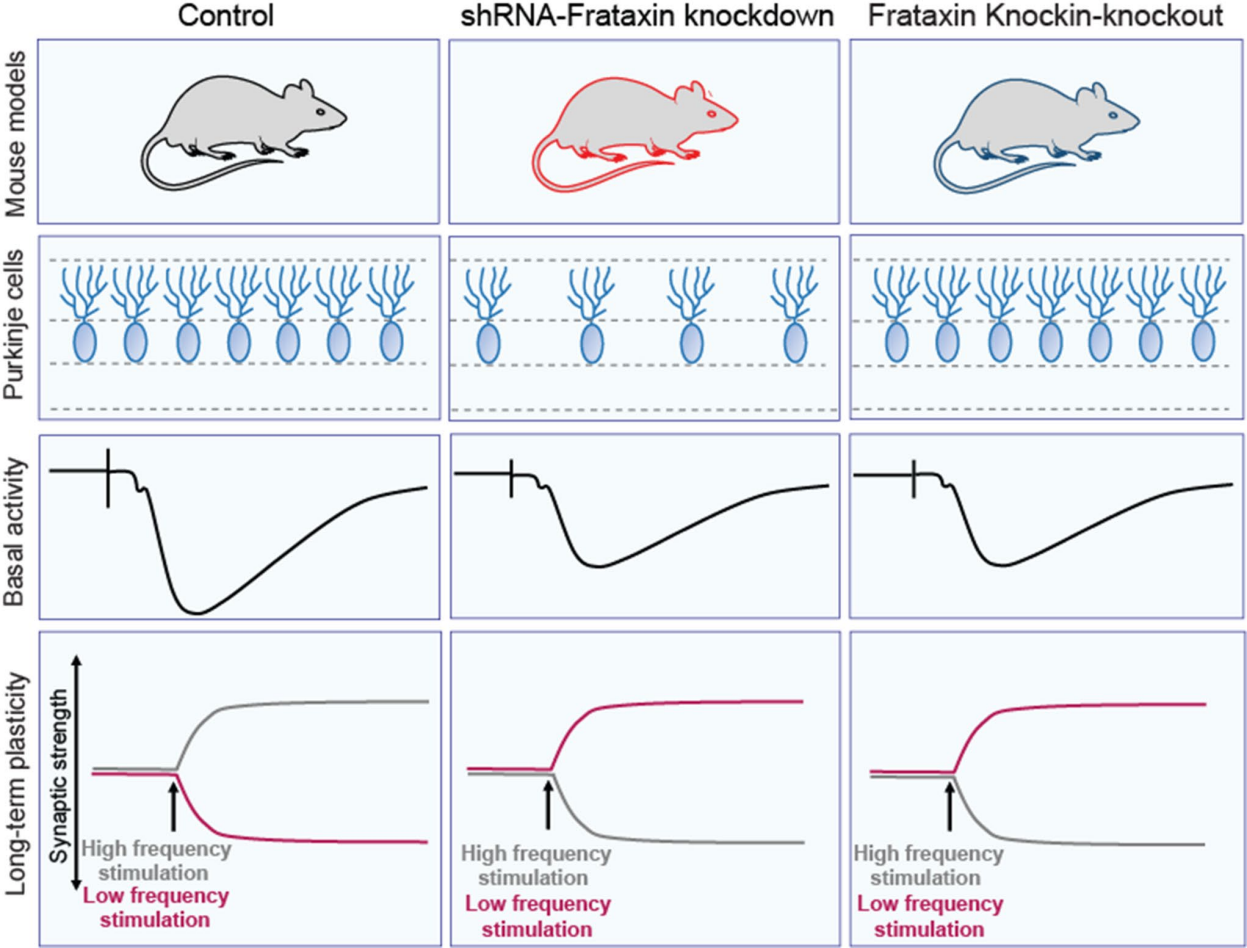

**Supplementary Information:**

The online version contains supplementary material available at 10.1007/s12311-025-01796-0.

## Introduction

Friedreich ataxia (FRDA) is a multisystemic, life-shortening, autosomal recessive degenerative disorder caused primarily by biallelic GAA repeat expansions in the first intron of the *FXN* gene [1, 2]. These repeats transcriptionally repress the gene and pathologically reduce levels of the ubiquitously expressed frataxin (FXN) protein [3, 4]. A small number of patients have compound heterozygous variants with an expansion on one allele in conjunction with a point mutation or deletion. FXN facilitates the assembly of iron-sulfur clusters (Fe/S) and is involved in other metabolic processes [5, 6] leading to disrupted biogenesis of Fe/S proteins with associated mitochondrial and cellular dysfunction [5, 7, 8]. A number of mouse models have been developed to investigate the molecular and cellular mechanisms of FRDA [9, 10]. Despite significant progress in the understanding of disease etiology in these mouse models, the underlying neurological targets of FRDA remain incompletely understood, limiting the ability to direct recent advances in gene therapy to specific neuroanatomical locations.

Identification of the anatomical substrates of gait and limb ataxia is not fully elucidated. Previously, neurologists have attributed these motor symptoms to early degeneration of the large dorsal root ganglion cells and their axons in the dorsal columns, with later loss of the excitatory neurons in the dentate nucleus and their efferent fibers in the superior cerebellar peduncles [1, 11, 12, 13]. More recent autopsy studies demonstrate significant pathological aberrations in PC morphology without cell death during later disease stages [1, 14, 15], and functional magnetic resonance imaging (fMRI) data from FRDA patients reveal significant functional pathology in the cerebellar cortex but also some evidence of PC changes based on enlargement of cerebellar folia [16, 17, 18]. To account for involvement of cerebellar cortex, the pathophysiological mechanisms of FRDA could include several components: (1) cell type-specific vulnerability despite the ubiquitous reduction of FXN in all cells, (2) an initial pathology of cellular dysfunction rather than death), and (3) ongoing circuit dysfunction in the cerebellar cortex as a crucial anatomical locus. Understanding how such events contribute to symptomatology has important therapeutic consequences, particularly for PCs as the sole output neurons of the cerebellar cortex [1, 14, 19]. Here, we examine the contribution of PCs to the neuropathological mechanism of FRDA by investigating the hypothesis that FXN deficiency leads to bio-physiological dysfunction and structural abnormalities in PC in symptomatic disease stages in FRDAkd and KIKO FRDA mouse models.

## Materials and Methods

### Human Cerebellar Tissue

Human cerebellar sections were obtained from the autopsy service of the Children’s Hospital of Philadelphia (CHOP) from four patients: (1) a 12-year-old female with cystic fibrosis who died during lung transplant surgery, (2) a 10-year-old male with coarctation of the aorta who died undergoing surgical repair, (3) a 14-year-old male who died of abdominal aortic dissection, and (4) a previously healthy 3-year-old male who died of pneumonia. The use of patient tissue and experimental procedures were approved by the Children’s Hospital of Philadelphia Institutional review board and performed in accordance with the Declaration of Helsinki.

### Nonhuman Primate Cerebellar Tissue

Nonhuman primate control tissue was obtained from animals housed in stainless steel squeeze-back cages at the University of Pennsylvania Nonhuman Primate Research Program facility, accredited by the Association for Assessment and Accreditation of Laboratory Animal Care. Animals received varied enrichments such as food treats, visual and auditory stimuli, manipulatives, and social interactions. Upon scheduled necropsy events, the brains from naïve cynomolgus or rhesus macaques were promptly collected following euthanasia and immersed in 10% neutral buffered formalin for 24 h. The brains were then prepared in 4 mm coronal slices using a brain blade and placed into cassettes [20]. The trimmed slices were allowed to fix for another day, placed in 70% ethanol, processed in a Leica ASP300S tissue processor, and embedded in paraffin blocks. Unstained slides were prepared from the blocks that contained the cerebellum.

### Frataxin Transgenic Mouse Models

All animals were treated in accordance with relevant guidelines and regulations outlined in the National Institutes of Health Guide for the Care and Use of Laboratory Animals and approved by the CHOP Institutional Animal Care and Use Committee. The shRNA knockdown or doxycycline-inducible FXN mice (FRDAkd) were originally obtained from Drs. Geschwind and Vijayedran at UCLA, then bred with C57BL/6J mice (The Jackson Laboratory Stock No: 000664) to generate equal proportions of wild-type and FRDAkd mice [10]. The FRDAkd mouse contains a doxycycline inducible shRNA that markedly decreases frataxin expression (to < 5% of control) on doxycycline administration. The knock-in/ knock-out (KIKO) mice were from the Jackson Laboratory (B6. Cg-Fxntm1.1Pand Fxntm1Mkn/J; stock number 012329) and were bred with C57BL/6 line from Charles River Laboratories. The KIKO line contains a knock in of one allele of the FXN (GAA)230 expansion mutation on one chromosome and a FXN allele with an exon-4 deletion on the homologous chromosome [9], leading to frataxin levels of 25–30% of control. KIKO mice were tested at 16–18 months of age, with age-matched mice harboring a knock in GAA of 230 in combination with a wildtype FXN allele (KIWT, equivalent to a human carrier) used as littermate controls. To induce FXN knockdown in FRDAkd mice, cohorts of transgenic mice at 4 months old were fed a doxycycline-compounded chow diet (200 PPM doxycycline (Animal Specialties and Provisions, LLC., Quakertown, PA, United States) for 16–18 weeks. Age-matched FRDAkd mice that received a regular chow diet (Uninduced FRDAkd) were used as controls. All mice were maintained in the CHOP vivarium and genotyped at weaning by a commercial vendor (Transnetyx, Cordova, TN, United States), and both sexes were used in all experiments.

### Histological Analysis

Mice were anesthetized and transcardially perfused first with 1X phosphate buffered saline (PBS) followed by 4% paraformaldehyde. The brains were removed and postfixed overnight in 4% paraformaldehyde, washed in PBS, dehydrated in 30% sucrose, and embedded in paraffin. 5 μm thick sagittal sections were cut on a microtome. Tissue pathology was assessed by immunohistochemical analysis using methods we have previously reported [21, 22]. Briefly, paraffin-embedded sections were deparaffinized, rehydrated, and antigen-retrieved in antigen unmasking solution (Vector Laboratories). Sections were then blocked (5% normal goat serum, 1% bovine serum albumin in 0.3% Triton X-100 in PBS) at room temperature for 1 h and then incubated with primary antibodies (frataxin, postsynaptic density protein 93, and calbindin D28K) at 4◦C overnight. The following primary antibodies were used: rabbit frataxin (FXN, 1:250; Abcam 17402), mouse Calbindin DK28K (CB, 1:50; Abcam 82812), rabbit Calbindin D-28 K (1:100; Proteintech 14479-1-AP), and mouse postsynaptic density protein 93 (PSD93,1:100; Neuromab 75–284). The cerebellar sections were washed three times with 1X PBS before incubation in secondary antibodies conjugated to Alexa fluor 488 (1:500; Invitrogen, A11034) or 568 (1:500; Invitrogen, A11031) at room temperature for 60–90 min. Following three washes with PBS, slides were mounted with Vectashield containing 4’,6-diamidino-2-phenylindole (DAPI, Vector Laboratories, H-1200) and imaged using a Leica epifluorescence microscope. PC density was measured by scanning the entire length of the PC layer in cortical cerebellar cortex. PCs were identified based on morphology, location, and expression of CB and PSD93. Considering the size difference between the sections and plane of section, the PC layer was scanned and measured using Image J software (https://imagej.nih.gov/ij/) to obtain the number of PCs per standardized unit length (µm).

### Western Blots

Mitochondrial protein expression in the cerebellum from the FRDAkd mouse was assessed as previously described [23]. Protein concentration was determined using the bicinchoninic acid (BCA) protein assay kit (Thermo Fisher Scientific, 23225). 20–30 micrograms of proteins were loaded into a 4–12% NuPAGE sodium dodecyl sulfate (SDS) polyacrylamide gel. After electrophoresis, the proteins were transferred to a nitrocellulose membrane and incubated with primary antibodies [FXN (1:500; Abcam ab175402)], Translocase of outer mitochondrial membrane 20 (TOMM20, 1:1000; Abcam 78547), 75-kDA glucose-regulated protein (GRP75,1:1000; Abcam 2799), mitochondrial transcription factor A (TFAM, 1:1000; Abcam 13607), Total OXPHOS Cocktail (1:500; Abcam ab110413), actin (1:5000; Abcam 3280), and Ca^2+^/calmodulin-dependent protein kinase II (CAMKII, 1:1000; Invitrogen MAI-048)] overnight at 4 °C. The Total OXPHOS Cocktail contains the following mitochondrial markers: ATP synthase F1 subunit alpha (ATP5A), ubiquinol cytochrome b-c1 complex subunit 2 (UQCRC2), mitochondrially encoded cytochrome c oxidase I (MTCO1), and succinate dehydrogenase (SDHB), NADH dehydrogenase [ubiquinone] 1 beta subcomplex subunit 8 (NDUFB8). Following 1^o^ antibody incubation, the blots were washed three times with TBS-T and incubated in secondary mouse or rabbit antibody (1:2000 Cell Signaling Technology) using 3% skim milk diluted in Tween/Tris-buffered saline (TBS-T) for 1.5 h at room temperature. Blots were stripped with stripping buffer (2% S DS, 50 mM Tris, pH 6.8, and 100mM β-mercaptoethanol) for 45 min at room temperature between probing for different proteins. Bands obtained were quantified using ImageJ software and normalized to loading control actin.

### Electrophysiology

*Cerebellar slice preparation and maintenance*: Cerebellar slices from 16 to 18 months old KIKO or approximately 8 months old FRDAkd (Treated for 16–18 weeks beginning at 4 months of age) mice were obtained by anesthetizing with isoflurane then transcardially perfusing with iced cold (0–4 °C) choline cutting solution containing in (mM): Choline-Cl 120, KCl 2.5, CaCl_2_ (Anhydrous) 0.5, MgSO_4_ (Anhydrous) 10, NaH_2_PO_4_ 1.25, choline-HCO_3_ 20, and glucose 25, HEPES 20, Na-Ascorbate 5, Na-Pyruvate 3, and Thiourea 2. The whole brain was rapidly removed and immersed in ice-slush choline cutting solution for 2–3 min. Transverse or sagittal cerebellar slices (250 μm) were cut in the choline cutting solution using the Leica VT1200S vibratome. Prior to recordings, slices were allowed to recover at 32ºC in recovery aCSF (115 mM NaCl, 2.5 mM KCl, 1mM MgSO_4_, 2.5 mM CaCl_2_, 1.4 mM NaH_2_PO_4_, 30.8 mM NaHCO_3_, 15.5 mM glucose, 5mM NaAscorbate, 3mM NaPyruvate, and 2mM Thiourea) for 2 h prior to recordings. Following recovery, slices were transferred to a submerged chamber (RC-27, Warner Instruments) and were continuously perfused with oxygenated recording aCSF (128mM NaCl, 2.5 mM KCl, 1mM MgSO_4_, 2.5mM CaCl_2_, 1.4mM NaH_2_PO_4_, 30mM NaHCO_3_, 15.5mM glucose) with a flow rate of 2 mL/min using a gravity-driven perfusion system. All aCSF and cutting solutions were equilibrated with 95% O_2_/5% CO_2_ to maintain a pH near 7.4 and all recordings were performed at 32 °C.

*Field recordings*: Parallel fiber-PC (PF-PC) field potentials were recorded on submerged transverse or parasagittal slices (250 μm) covered with a continuous flow of carbogenated (95% O_2_/5% CO_2_) aCSF (2 ml/min) at 34ºC. Cortical cerebellar structures were easily visualized for placement of stimulating and recording electrodes using a 4x objective and an IR-sensitive CCD camera (Rolera, Qimaging) attached to a BX-51WI microscope (Olympus, USA). For electrical stimulation of the PFs), a tungsten concentric bipolar stimulating electrode (FHC Scientific) was placed in the middle of the molecular layer. Evoked field potentials were recorded with a borosilicate glass recording electrode (2–3 MΩ) filled with recording aCSF and placed in the molecular layer near the PC layer. The recording electrode was placed approximately 400 μm away from the stimulating electrode and at the depth in the slice that gave the largest field excitatory post-synaptic potential (fEPSP) amplitude. Waveforms were acquired with a Multiclamp 700B amplifier (Molecular Devices, USA) connected to a Digidata 1440 A analog-digital converter (Molecular Devices, USA). Signals were sampled at 10 kHz and filtered with a 5 kHz low-pass Bessel filter. For construction of input-output (I-O) curves of synaptic strength, the PFs were stimulated at 0.1 Hz to minimize synaptic failures and the stimulation intensity ranged from 0 to 300µA in 20µA increments. To measure short term plasticity (STP) of dendritic fEPSPs, paired-pulse stimulation of the PF at different intervals (in ms: 10, 25, 50, 100, and 200) were applied and repeated 6–10 times for each interval at 0.1 Hz. Paired-pulse ratios (PPR) are given as the amplitude of response 2/amplitude of response 1. To record long-term potentiation (LTP) and long-term depression (LTD), stable baseline responses were collected at 0.033 Hz for 30 min and post-induction responses were normalized to the final 10 min. LTP was induced a high frequency stimulation (HFS) protocol (4 trains of 500 msec at 200 Hz separated by 5 min interval) and post induction fEPSPs were recorded at 0.033 Hz for 60 min. LTD was induced by paired-pulse low frequency stimulations (PP-LFS) separated by either 50 [α-amino-3-hydroxy-5-methyl-4-isoxazolepropionic acid receptor (AMPAR) dependent] or 200ms [N-methyl-D-aspartate receptor (NMDAR) dependent] [24] delivered at 1 Hz. For the recordings of PPR, LTP, and LTD, the stimulus intensity was adjusted to half-maximal fEPSP noted during the construction of I-O curves. fEPSPs were quantified by measuring the slope of the linear part of the rising phase using Clampfit 10.7 (Molecular Devices, USA).

### Statistical Analysis

Electrophysiological traces were displayed off-line with the Clampfit software pClamp version 10.7 (Molecular Devices) and data points were extracted and then organized in Excel (Microsoft). Graphical plots were generated and analyzed using Prism 9 (GraphPad). In most cases, Student’s *t* test was used with genotype as the variable. Statistical significance for input-output relationship curves was determined by the Two-way analysis of variance (ANOVA) test followed by Sidak’s post hoc test. All data are given as the means ± SEM, with n being the number of cells/slices or animals. For all statistical tests, significance with respect to the control is indicated on the figures using the following symbols: *p < 0.05, **p < 0.01, ***p < 0.001, and ****p < 0.0001. Sample sizes were not predetermined by power analysis, but they conformed to those in similar studies. Data distribution was assumed to be normal.

## Results

### Histological Localization of FXN in Cerebellum

**Fig. 1:**
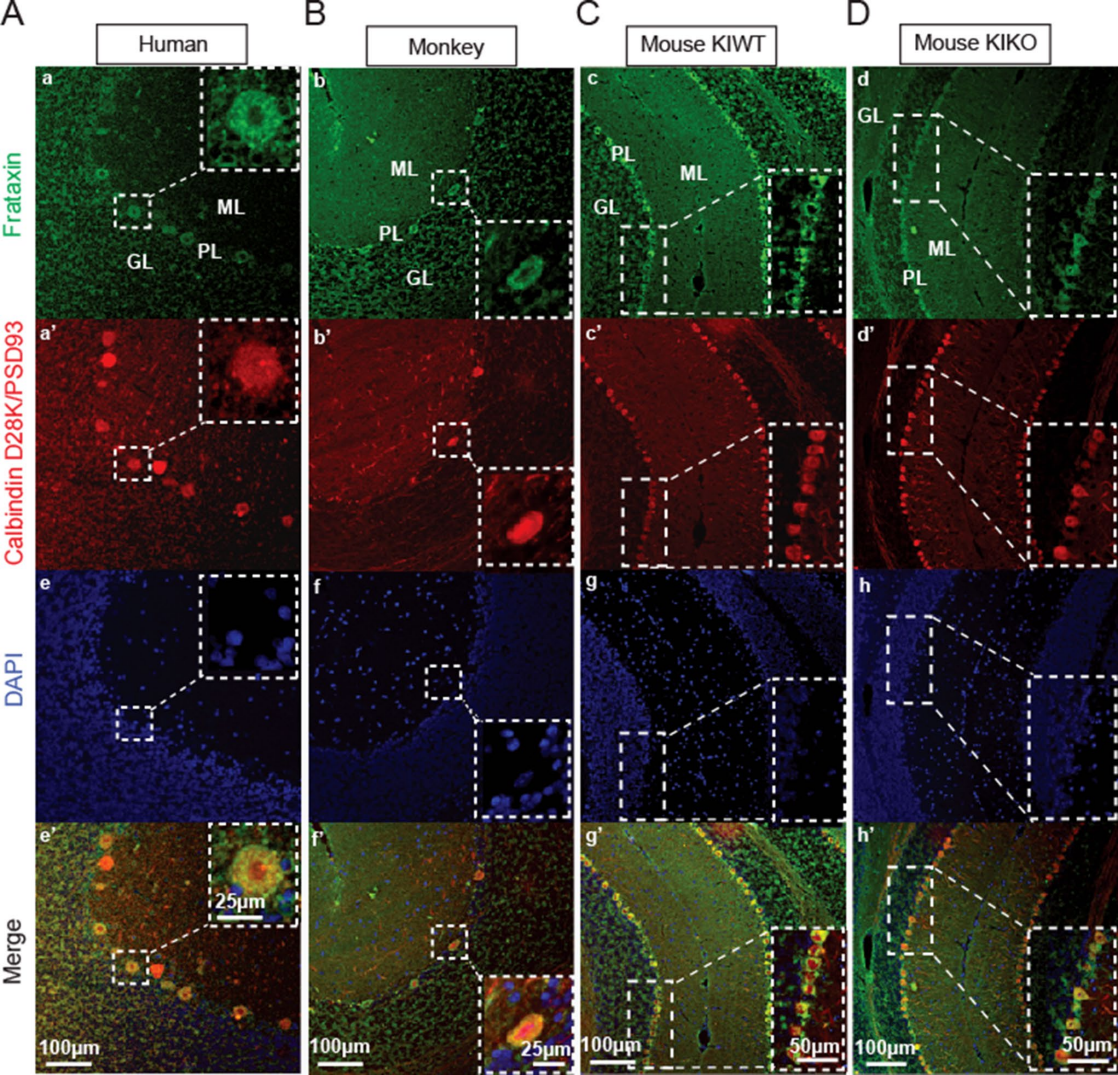
Frataxin expression in Purkinje cells is conserved across species. Representative micrographs of Human (**A**), Monkey (**B**), KIWT (**C**), and KIKO (**D**) tissues showing frataxin co-labeling with calbindin D28K or PSD93. (**a-d**) cerebellar sections stained with frataxin (green) (a’) human stained with PSD93 (red) (b’-d’) monkey and mice sections stained with calbindin D28 (Red) markers of Purkinje cells. (**e-h**) DAPI shows the presence of other cells in the GL = granule layer and ML = molecular layer. (e’-h’) Merged

While FXN is found in all cells and is deficient in all cells in FRDA, its expression level varies in different neurons. In mouse, monkey, and human, cerebellar cortical FXN levels are high in PCs by immunofluorescence (Fig. 1). Lower levels are found in granule cells and other cells in the cerebellar cortex. The similar distribution of frataxin across species suggests that mice provide a suitable animal to study cerebellar circuit dysfunction from FXN deficiency.

### Changes in Mitochondrial Protein Levels in the Cerebellum of FRDA Mice

**Fig. 2:**
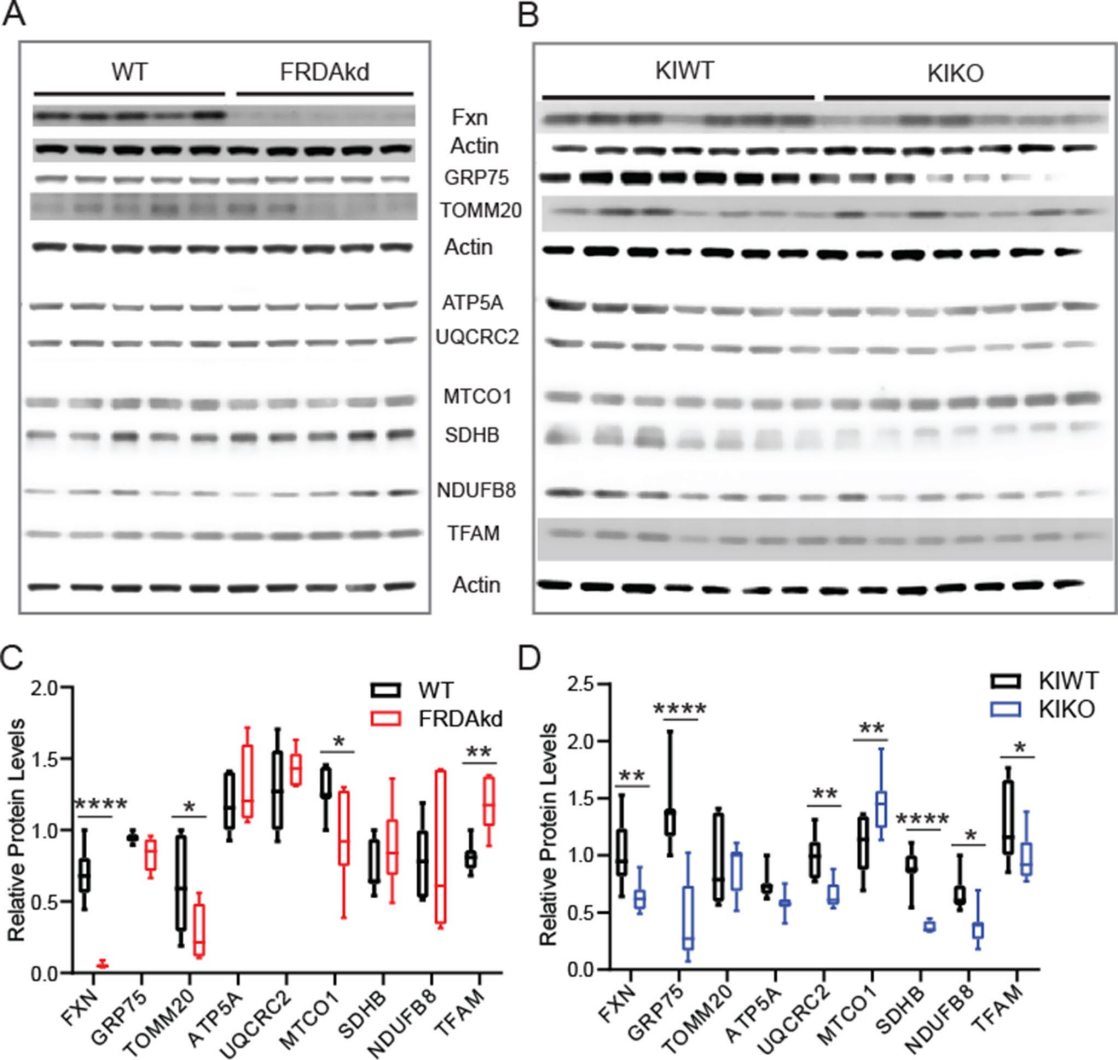
Mitochondrial proteins levels are dysregulated in the cerebellum of FRDA mice. (**A & B**) Representative micrograph of Western blot of mitochondrial biogenesis proteins from FRDAkd (**A**) mice induced for 16 weeks and 16–18 months old KIKO mice (**B**). (**C & D**) Quantitative analysis plot of FXN, GRP75, TOMM20, ATP5A, UQCRC2, MTCO1, SDHB, NDUFB8, and TFAM normalized to internal control actin. Data are given as mean ± SEM and analyzed by two-way ANOVA followed by Bonferroni *post hoc* test. *P < 0.05, **P < 0.01, ***P < 0.001, ****P < 0.0001; WT, FRDAkd: *n* = 7, 6, F_(8, 99)_ = 5.56; KIWT, KIKO: *n* = 7 for both, F_(8, 108)_ = 7.90

Mitochondrial dysfunction is a major pathophysiological hallmark of FRDA. Thus, we assessed mitochondrial health by analyzing the expression levels of the mitochondrial proteins FXN, GRP75, TOMM20, ATP5A, UQCRC2, MTCO1, SDHB, NDUFB8, and TFAM in both FRDA mouse models (Fig. 2A). These proteins are important in the maintenance of mitochondrial homeostasis and biogenesis. Quantitative analysis of Western blots from FRDAkd (Fig. 2A & C; WT, FRDAkd: *n* = 7, 6; *P <* 0.0001, F_(8, 99)_ = 5.56) and KIKO (Fig. 2B & D; KIWT, KIKO: *n* = 7, 7; *P <* 0.0001, F_(8, 108)_ = 7.90) mice revealed significant changes in the expression level of these mitochondrial markers. In the FRDAkd mice, expression of FXN (WT = 0.68 ± 0.07, FRDAkd = 0.05 ± 0.02, *P <* 0.0001), TOMM20 (WT = 0.61 ± 0.13, FRDAkd = 0.28 ± 0.08; *P =* 0.013), and MTCO1 (WT = 1.28 ± 0.06, FRDAkd = 0.94 ± 0.13, *P =* 0.012) markedly decreased, whereas that of TFAM increased (WT = 0.82 ± 0.04, FRDAkd = 1.18 ± 0.08; *P =* 0.007). GRP75 (WT = 0.95 ± 0.01, FRDAkd = 0.83 ± 0.05; *P =* 0.392), ATP5A (WT = 1.17 ± 0.07, FRDAkd = 1.31 ± 0.11; *P =* 0.328), UQCRC2 (WT = 1.28 ± 0.11, FRDAkd = 1.44 ± 0.05; *P =* 0.256), SDHB (WT = 0.75 ± 0.07, FRDAkd = 0.88 ± 0.12; *P =* 0.357), and NDUFB8 (WT = 0.76 ± 0.10, FRDAkd = 0.79 ± 0.21; *P =* 0.851) were unaffected in the FRDAkd mice. As in the FRDAkd mice, the expression of FXN was reduced in KIKO mice (KIWT = 0.94 ± 0.08, KIKO = 0.55 ± 0.07, *P =* 0.003);similar reduced expression was also observed for GPR75 (KIWT = 1.37 ± 0.13, KIKO = 0.44 ± 0.13, *P <* 0.0001), UQCRC2 (KIWT = 0.99 ± 0.07, KIKO = 0.65 ± 0.04, *P =* 0.007), SDHB (WT = 0.88 ± 0.07, FRDAkd = 0.37 ± 0.02, *P <* 0.0001), NDUFB8 (KIWT = 0.67 ± 0.06, KIKO = 0.39 ± 0.06, *P =* 0.025), as well as TFAM (KIWT = 1.26 ± 0.13, KIKO = 0.97 ± 0.08, *P =* 0.022). By contrast, the expression level of MTCO1 (KIWT = 1.09 ± 0.09, KIKO = 1.44 ± 0.10, *P =* 0.005) significantly increased in those KIKO mice. TOMM20 (KIWT = 0.92 ± 0.13, KIKO = 0.88 ± 0.08, *P =* 0.806) and ATP5A (KIWT = 0.74 ± 0.05, KIKO = 0.58 ± 0.04, *P =* 0.199) were unaffected in the KIKO mice. Taken together, these results revealed significant changes in the expression of important mitochondrial proteins, albeit without a systematic or concordant pattern between the two FRDA mouse models.

### PC Survival in FRDA Mice

**Fig. 3:**
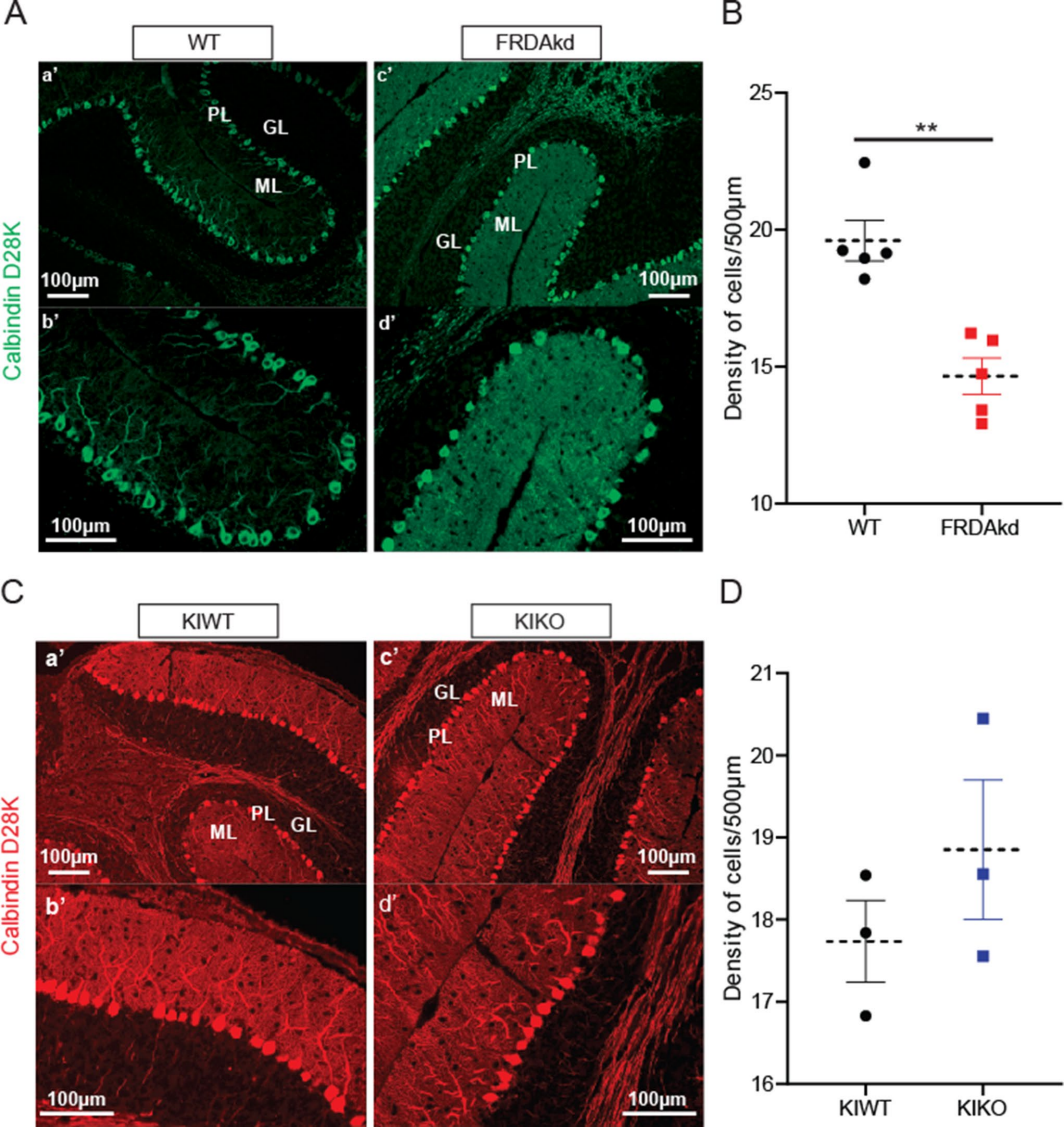
Disparate effect of FXN loss on Cerebellar Purkinje cell density. Representative micrographs of FRDAkd (**A**) and KIKO (**C**) in the cerebellum. (**a**,** b**) Control mice and FRDAkd (**c**,** d**) stained with calbindin D28K; KIWT (a’,b’) and KIKO (c’,d’) with calbindin D28 (red) (**B**) Quantification of calbindin D28-positive Purkinje neurons in control mice and FRDAkd mice and (**D**) in KIKO and KIWT mice. Approximately 30 images per mouse were analyzed by a blinded individual. Data are given as mean ± SEM and analyzed by 2-tailed unpaired Student’s *t* test. **P < 0.01, Control (uninduced FRDAkd), FRDAkd (Induced): *n* = 5 for both; KIWT, KIKO: *n* = 3 for both

While PC loss is traditionally low in human FRDA, FRDAkd mice treated with Dox for 18 weeks showed a moderate reduction in the number of cells expressing the PC marker mitochondrial α-F1‐ATP synthase [23]. We confirmed these observations in induced FRDAkd mice of the same age and treatment duration using CB, another marker of PCs (Fig. 3A-B; WT = 19.6 ± 0.73, FRDAkd = 14.6 ± 0. 0.66; *P* = 0.001, *n* = 5 for each genotype). In contrast, CB positive PCs in the KIKO mouse model at 15 months of age, as in human FRDA, are relatively preserved (Fig. 3C-D; WT = 17.7 ± 0.50, FRDAkd = 18.8 ± 0.85; *P* = 0.32, *n* = 3 for each genotype), showing that loss of FXN differentially influences PC survival in FRDAkd and KIKO mice.

### Basal Synaptic Transmission Impairments

**Fig. 4:**
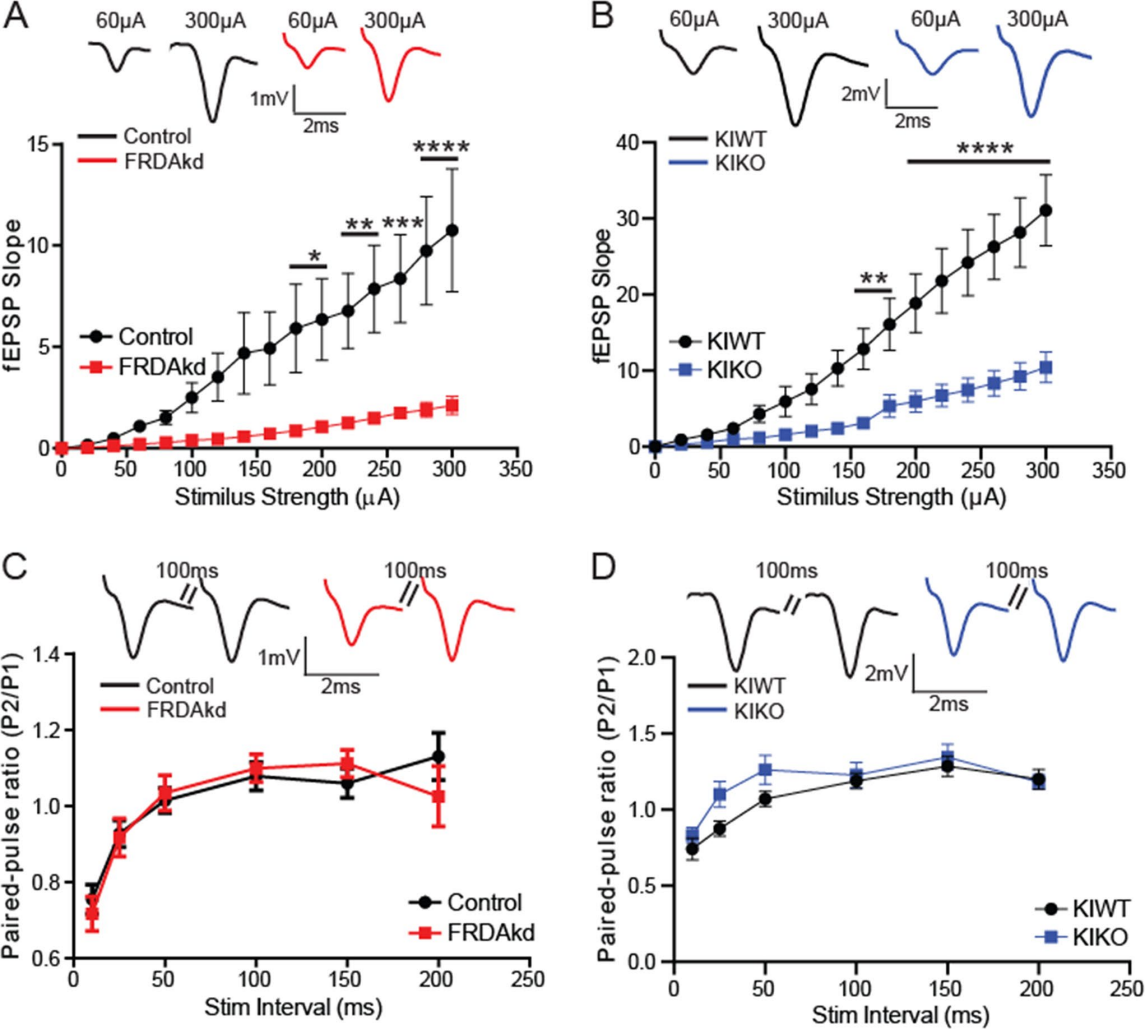
Synaptic transmission at parallel fiber-Purkinje cell synapses is impaired in FRDA mice. (**A**) I-O relationship of molecular layer fEPSP in control (Uninduced FRDAkd) and induced FRDAkd (16–18 weeks) mice. *P < 0.05, **P < 0.01, ***<0.001, ****P < 0.0001; Control, FRDAKD: *n* = 20/10, 24/12, slices/mice; F_(1, 654)_ = 93.6. *Top traces*: Representative waveforms of fEPSP at low and high stimulation amplitudes. (**B**) I-O relationship of molecular layer fEPSP in KIWT and KIKO mice (16–18 months old). **P < 0.01, ****P < 0.0001; KIWT, KIKO: *n* = 20/10, 30/15, slices/mice; F_(1, 766)_ = 170.3. *Top traces*: Representative waveforms of fEPSP at low and high stimulation amplitudes. (**C**) Short-term plasticity of fEPSP measured at different paired-pulse intervals in control and induced FRDAkd mice. Control, FRDAkd: *n* = 59/30, 46/23, slices/mice; *P* > 0.05, F_(1, 618)_ = 0.14. *Top traces*: Representative waveforms of fEPSP at 100ms paired-pulse interval. (**D**) Short-term plasticity of fEPSP measured at different paired-pulse intervals in KIWT and KIKO mice. KIWT, KIKO: *n* = 35/18, 42/21, slices/mice; *P* > 0.05, F_(1, 450)_ = 5.72). *Top traces*: Representative waveforms of fEPSP at 100ms paired-pulse interval. Data are given as mean ± SEM and analyzed by Two-way ANOVA followed by Sidak *post hoc* test. I-O relationships were constructed by increasing stimulus intensity from 0-300µA in 20µA increment

Given the non-systematic changes in mitochondrial protein levels and the modest difference in PC density in the two mouse models, synaptic deficits at PCs might link the two models to disease phenotype. To that end, we recorded field synaptic transmission at the molecular layer of the cerebellar cortex in response to stimulation of PFs. Stimulation of these PFs always resulted in a very fast inward field excitatory postsynaptic potentials (fEPSPs) as previously noted at PF-PC synapses in the cerebellum [25, 26]. Examining the efficacy of basal synaptic transmission at increasing amplitude intensities, the fEPSP I-O relationship curve was significantly reduced in induced FRDAkd mice compared to controls (FRDAkd mice on normal chow), beginning at stimulus intensity of 180µA (Fig. 4A; Control, FRDAkd: *n* = 20/10, 24/12, slices/mice; *P* < 0.0001, (F_(1, 654)_ = 93.6). Similarly, the I-O curve was reduced in KIKO mice compared to KIWT controls beginning at 160µA (Fig. 4B, KIWT, KIKO: *n* = 20/10, 30/15, slices/mice; *P* < 0.0001, F_(1, 766)_ = 170.3). The paired-pulse ratio (PPR) of fEPSPs, a measure of presynaptic function and short-term plasticity, in both induced FRDAkd (Fig. 4C; Control, FRDAkd: *n* = 59/30, 46/23, slices/mice; *P* > 0.05, F_(1, 618)_ = 0.14) and KIKO (Fig. 4D; KIWT, KIKO: *n* = 35/18, 42/21, slices/mice; *P* > 0.05, F_(1, 450)_ = 5.72) mice was indistinguishable from controls at all the ISI times tested. Taken together, these results suggest both FRDA models exhibited concordant reduction in synaptic connectivity despite the likely absence of presynaptic dysfunction.

### Impairments of LTP and LTD Induced by a Short Interval Paired Pulse Protocol

**Fig. 5:**
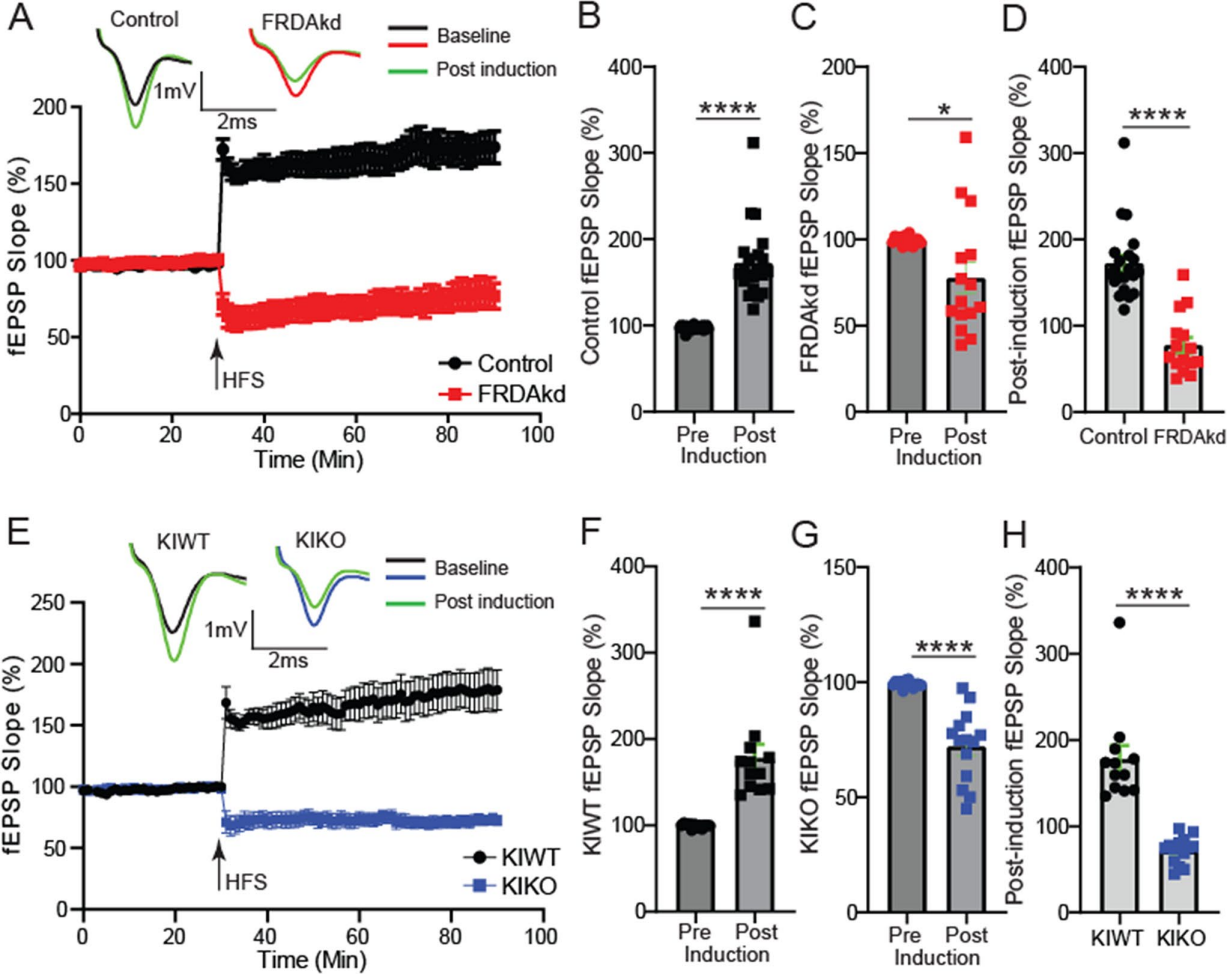
Concordant impairments of LTP in FRDAkd and KIKO mice. (**A**) Time course of Parallel fiber-Purkinje cell synapse fEPSP slope 30 min before and 60 min after a train of high frequency stimulation LTP protocol in control and FRDAkd mice. Top traces: representative traces of fEPSP recorded at baseline and after LTP induction. (**B**,** C**) Histograms of percent changes in fEPSP following LTP induction in control (**B**) and FRDAkd (**C**) mice relative to baseline. (**D**) Comparative analysis of percent change in post-induction fEPSP magnitude between control and FRDAkd mice. (**E**) Time course of Parallel fiber-Purkinje cell synapse fEPSP slope 30 min before and 60 min after the train of high frequency stimulation LTP protocol in KIWT and KIKO mice. Top traces: representative traces of fEPSP recorded at baseline and after LTP induction. (**F**,** G**) Histograms of percent changes in fEPSP following LTP induction in KIWT (**F**) and KIKO (**G**) mice relative to baseline. (**H**) Comparative analysis of percent change in post-induction fEPSP magnitude between KIWT and KIKO mice. The last 10 min of baseline and/or post-induction timelines were used for all comparative analyses. *P < 0.05, ****P < 0.0001; Control, FRDAKD: *n* = 21/21, 15/15, slices/mice; KIWT, KIKO: *n* = 12/12, 14/14, slices/mice. Data are given as mean ± SEM and analyzed by the 2-tailed unpaired Student’s t tests

Long-term plasticity mechanisms at PF-PC synapses, such as LTD and LTP, provide cellular correlates of cerebellar motor learning [27]. Therefore, we determined if PF-PC synapses in both KIKO and FRDAkd mice could undergo those long-term adaptive processes. A long HFS protocol (4 × 200 Hz with 5 min intervals) [28] delivered to the PF in the molecular layer of the cerebellum in uninduced FRDAkd mice reliably induced LTP, noted by comparison of averaged baseline fEPSP slope measured from last 10 min of a stable 30 min recording to the averaged fEPSP slope from the last 10 min of a 60 min post-tetanus time course (Figs. 5A-B and 75.23 ± 9.32%; *P* < 0.0001, *n* = 21/21, slices/mice). Unexpectedly, this HFS protocol induced a modest depression of the fEPSP slope in the FRDAkd mice rather than potentiation (Fig. 5C and 22.0 ± 8.96%; *P* = 0.021, *n* = 15/15, slices/mice). As expected from the opposite trajectories of the post-tetanus response in FRDAkd and control mice, the post-tetanus responses of the last 10 min were significantly reduced in the magnitude of the fEPSP slope in FRDAkd mice (Fig. 5D; -95.1 ± 13.4%; Control, FRDAkd: *n* = 21/21, 15/15, slices/mice; *P* < 0.0001). As in the un-induced FRDAkd mice, application of the HFS prominently potentiated the post-tetanus fEPSP response in KIWT mice (Figs. 5E-F and 78.7 ± 15.6%; *P* < 0.0001, *n* = 12/12, slices/mice). Application of the HFS protocol in KIKO mice significantly depressed of the fEPSP following tetanus as observed in the induced FRDAkd mice (Fig. 5G; -27.0 ± 4.2% below the baseline set at 100%; *P* < 0.0001, *n* = 14/14, slices/mice). Similarly, the post-tetanus response between KIWT and KIKO during the last 10 min of fEPSP timeline was significantly reduced in the magnitude of the fEPSP slope response in the KIKO mice (Fig. 5H; -105.8 ± 15.1%; KIWT, KIKO: *n* = 12/12, 14/14, slices/mice; *P* < 0.0001). These results suggest the FRDA models exhibited concordant defects in LTP despite the disparate effects on PC survival.

**Fig. 6:**
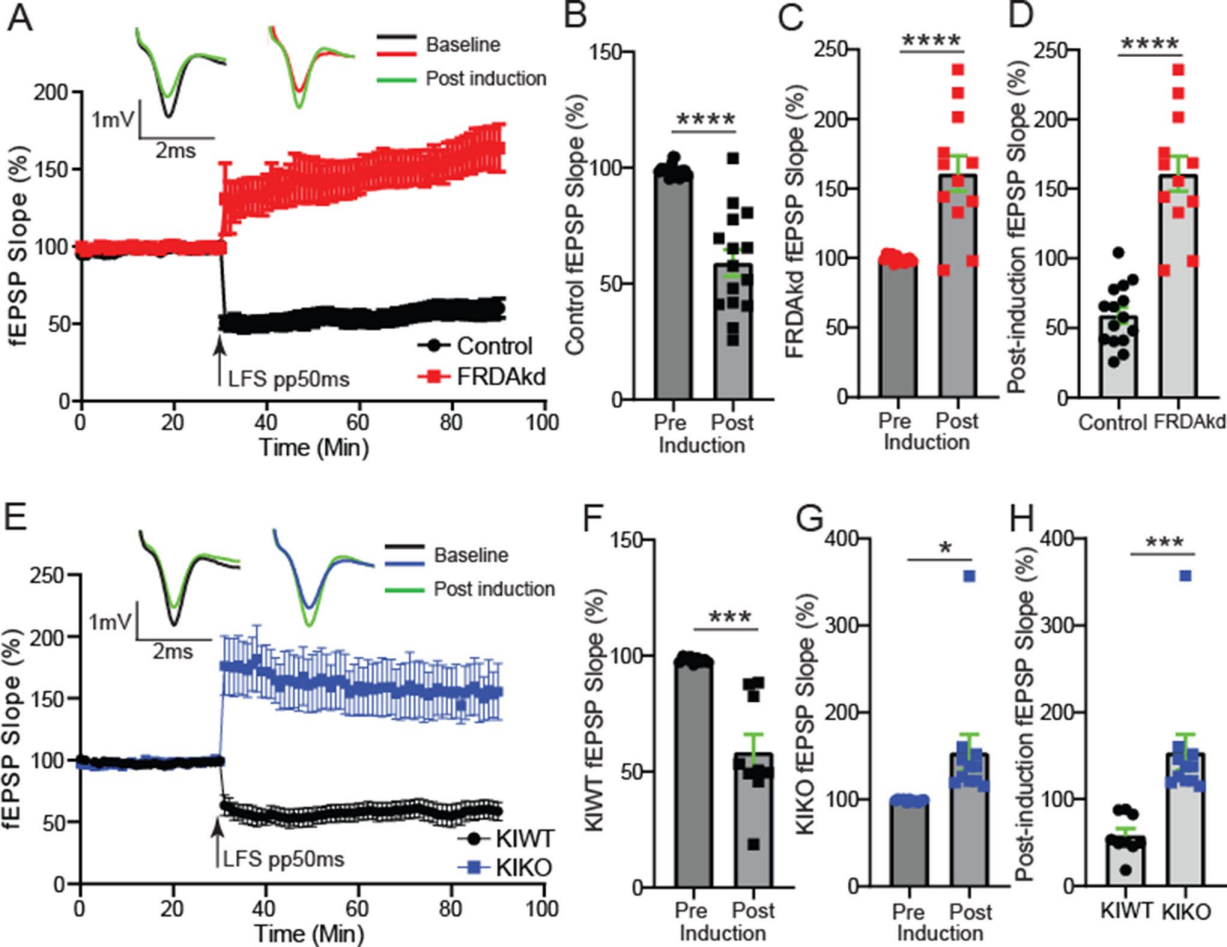
FRDA mice display similar impairments in AMPAR-dependent LTD. (**A**) Time course of Parallel fiber-Purkinje cell synapse fEPSP slope 30 min before and 60 min after a train of paired stimuli of 50ms interval delivered at 1 Hz for 15 min in control and FRDAkd mice. Top traces: representative traces of fEPSP recorded at baseline and after LTD induction. (**B**,** C**) Histograms of percent changes in fEPSP following LTD induction in control (**B**) and FRDAkd (**C**) mice relative to baseline. (**D**) Comparative analysis of percent change in post-induction fEPSP magnitude between control and FRDAkd mice. (**E**) Time course of Parallel fiber-Purkinje cell synapse fEPSP slope 30 min before and 60 min after the train of low frequency 50ms paired stimulation protocol in KIWT and KIKO mice. Top traces: representative traces of fEPSP recorded at baseline and after LTD induction. (**F**,** G**) Histograms of percent changes in fEPSP following LTD induction in KIWT (**F**) and KIKO (**G**) mice relative to baseline. (**H**) Comparative analysis of percent change in post-induction fEPSP magnitude between KIWT and KIKO mice. The last 10 min of baseline and/or post-induction timelines were used for all comparative analyses. *P < 0.05, ***P < 0.001, ****P < 0.0001; Control, FRDAkd: *n* = 15/15, 12/12, slices/mice; KIWT, KIKO: *n* = 9/9, 11/11, slices/mice. Data are given as mean ± SEM and analyzed by the 2-tailed unpaired Student’s t tests

One Hertz stimulation of afferents with constant interstimulus intervals for 15 min (1 Hz, 900 stimuli) reliably induces LTD in cortical and hippocampal tissues of young animals [29]. However, in many studies, LTD is not as readily induced by such protocol in adult cortical or hippocampal structures [24]. Interestingly, application of PP-LFS protocols at relatively short and long duration intervals reliably induced LTD in young and adult cortical or hippocampal structures alike [24]. Here, we applied the short duration paired-pulse (50ms interval) protocol [24] to determine whether it can induce LTD in the cerebellum as well as whether loss of FXN impinges on its expression. This protocol is thought to induce an AMPA-receptor form of LTD [24] and can be assessed by delivering 900 paired-pulse stimuli to the PF in the molecular layer of the cerebellum at 1 Hz with interval of the paired stimuli set at 50ms (PP50ms-LFS) and recorded fEPSP near the PC-layer as in the basal synaptic and LTP experiments above. Using the same baseline to post-induction comparative approach as in the LTP experiments, PP50ms-LFS protocol reliably induced LTD in uninduced FRDAkd (Fig. 6A-B; -40.1 ± 5.72%; *P* < 0.0001, *n* = 15/15, slices/mice). However, this LFS protocol induced LTP in FRDAkd mice rather than LTD (Figs. 6C and 61.5 ± 12.7%; *P* < 0.0001, *n* = 12/12, slices/mice). The post-tetanus responses of the last 10 min in induced and uninduced FRDAkd mice were significantly potentiated in the magnitude of the fEPSP in the induced mice (Figs. 6D and 101.9 ± 12.9%; *P* < 0.0001, Control, FRDAkd: *n* = 15/15, 12/12 slices/mice). As expected, the PP50ms-LFS protocol induced a robust LTD in the KIWT mice (Fig. 6E-F; -39.71 ± 7.77%; *P* = 0.0001, *n* = 9/9, slices/mice). As in the induced FRDAkd mice, this LTD protocol induced a significant potentiation of the fEPSP in KIKO mice instead of a depression (Figs. 6G and 55.3 ± 20.8%; *P* = 0.015, *n* = 11/11, slices/mice). As expected from these observations, the post-tetanus response during the last 10 min of fEPSP timeline was significantly potentiated in the magnitude of the fEPSP slope response in KIKO mice compared to KIWT mice (Figs. 6H and 95.6 ± 24.1% below the baseline set at 100%; KIWT, KIKO: *n* = 9/9, 11/11, slices/mice; *P* = 0.0009).

### Locus of the of Long-Term Plasticity Deficits and Involvement of CaMKII

**Fig. 7:**
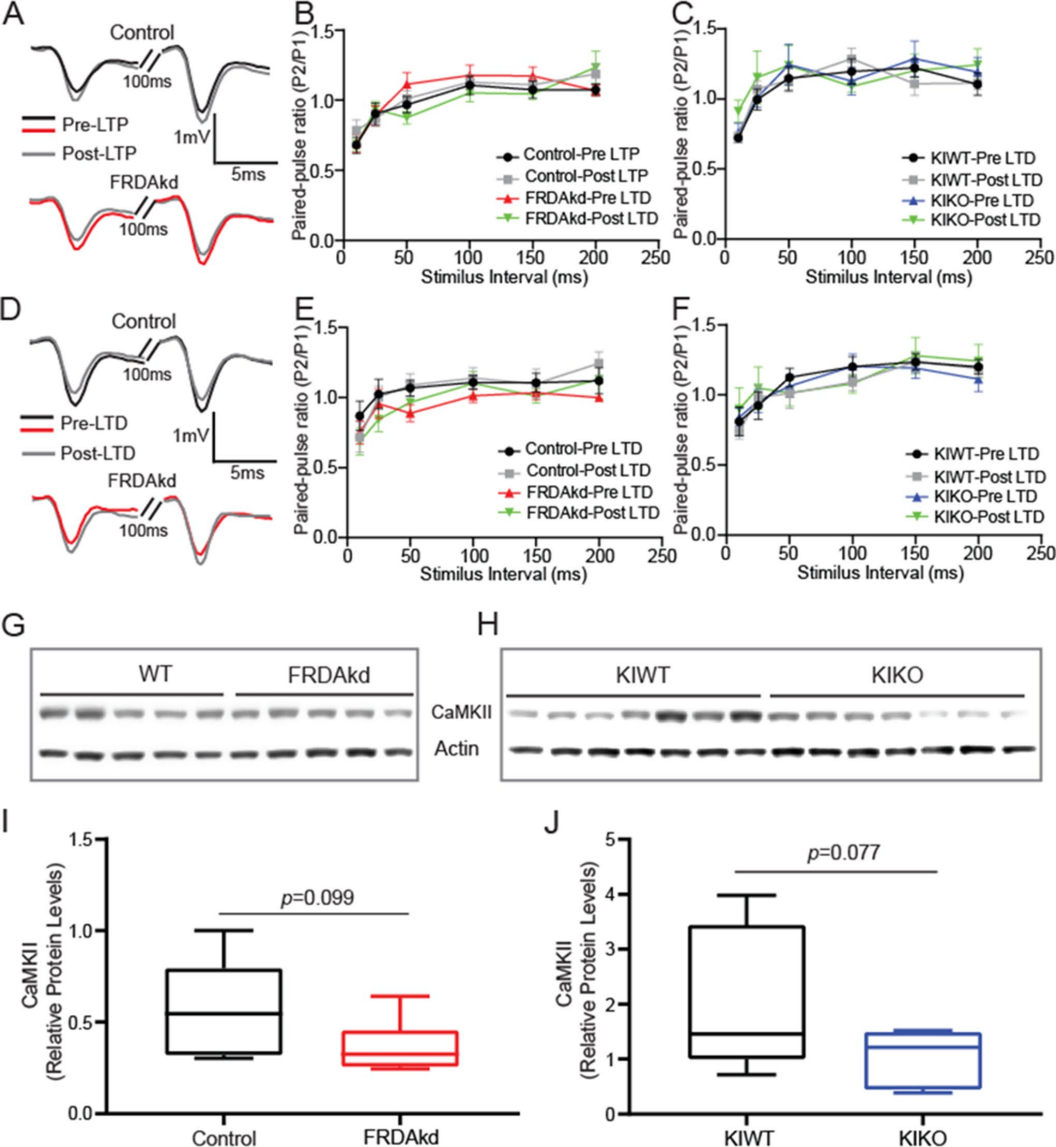
Postsynaptic localization of synaptic plasticity defects and preservation of CaMKII expression levels. (**A**) Example traces of paired fEPSPs before and after LTP induction in control (Top) and FRDAkd (Bottom) mice at 100ms stimulation intervals. (**B**) Paired-pulse ratio (fEPSP2/fEPSP1) measured at different intervals before and after LTP induction in control and FRDAkd mice. (**C**) Paired-pulse ratio (fEPSP2/fEPSP1) measured at different intervals before and after LTP induction in KIWT and KIKO mice. (**D**) Example traces of paired fEPSPs before and after LTD induction in control (Top) and FRDAkd (Bottom) mice at 100ms stimulation intervals. (**E**) Paired-pulse ratio (fEPSP2/fEPSP1) measured at different intervals before and after LTD induction in control and FRDAkd mice. (**F**) Paired-pulse ratio (fEPSP2/fEPSP1) measured at different intervals before and after LTD induction in KIWT and KIKO mice. For pre- and post-LTP PPR: Control, FRDAKD: *n* = 17/17, 15/15, slices/mice; KIWT, KIKO: *n* = 12/12, 14/14, slices/mice. For pre- and post-LTD PPR: Control, FRDAkd: *n* = 14/14, 12/12, slices/mice; KIWT, KIKO: *n* = 9/9, 11/11, slices/mice. (**G**,** H**) Representative micrograph of Western blot of CaMKII expression in FRDAkd (**G**) and KIKO (**H**). (**I**,** J**) Quantitative analysis plot of CaMKII levels in FRDAkd (**I**) and KIKO (**J**) mice normalized to internal control actin. Data are given as mean ± SEM and analyzed by analyzed by Two-way ANOVA (PPR data) or by the 2-tailed unpaired Student’s t tests (Western blotting data)

Different forms of long-term plasticity, either entirely postsynaptically or presynaptically expressed, have been observed at PF-PC synapses [30]. To investigate the locus of the long-term plasticity defects in FRDA mice, we compared PPR at stimulus intervals ranging from 10-200ms before and after induction. The PPR of fEPSPs in FRDAkd did not change after induction with the LTP protocol at any of ISI tested in both FRDAkd (Fig. 7A-B; Pre-LTP at 100ms ISI: Control = 1.11 ± 0.066 vs. FRDAkd = 1.18 ± 0.053, Post LTP at 100ms interval: Control = 1.13 ± 0.072 vs. FRDAkd = 1.12 ± 0.065; Control, FRDAkd: *n* = 17/17, 15/15, slices/mice; *P* = 0.35, F_(3, 360)_ = 1.10) and KIKO mice (Fig. 7C; Pre-LTP at 100ms ISI: KIWT = 1.20 ± 0.090 vs. KIKO = 1.29 ± 0.074, Post LTP at 100ms interval KIWT = 1.13 ± 0.099 vs. KIKO = 1.09 ± 0.039; KIWT, KIKO: *n* = 12/12, 14/14, slices/mice; *P* = 0.55, F_(3, 287)_ = 0.71). Similarly, the PPR was not altered by induction with the PP50 LTD protocol at any of ISI tested in either FRDAkd (Fig. 7D-E; Pre-LTD at 100ms ISI: Control = 1.11 ± 0.050 vs. FRDAkd = 1.14 ± 0.073, Post LTD at 100ms interval Control = 1.02 ± 0.045 vs. FRDAkd = 1.13 ± 0.069; Control, FRDAkd: *n* = 14/14, 12/12, slices/mice; *P* = 0.14, F_(3, 287)_ = 1.86) or KIKO mice (Fig. 7F; Pre-LTD at 100ms ISI: KIWT = 1.20 ± 0.069 vs. KIKO = 1.08 ± 0.064, Post LTD at 100ms ISI: KIWT = 1.20 ± 0.086 vs. KIKO = 1.09 ± 0.059; KIWT, KIKO: *n* = 9/9, 11/11, slices/mice; *P* = 0.78, F _(3, 216)_ = 0.36). To gain further mechanistic insights into the defective plasticity in FRDA mice, we measured levels of CaMKII by Western blotting. Quantitative analysis of these blots revealed a non-significant trend toward lower levels of CaMKII protein in both FRDAkd (Fig. 7G & I; WT = 0.58 ± 0.09, FRDAkd = 0.37 ± 0.06; *P* = 0.099; WT, FRDAkd: *n* = 7, 6) and KIKO (Fig. 7H & J; KIWT = 1.98 ± 0.48, KIKO = 0.97 ± 0.19; *P* = 0.077, KIWT, KIKO: *n* = 7, 7) mice. Altogether, these results suggest that the PC or post-synaptic terminal represents the main locus of the long-term plasticity deficits without a significant change in CaMKII levels from whole cerebellar lysates.

### Impairment of LTD Induced by a Long Interval Paired Pulse Protocol

**Fig. 8:**
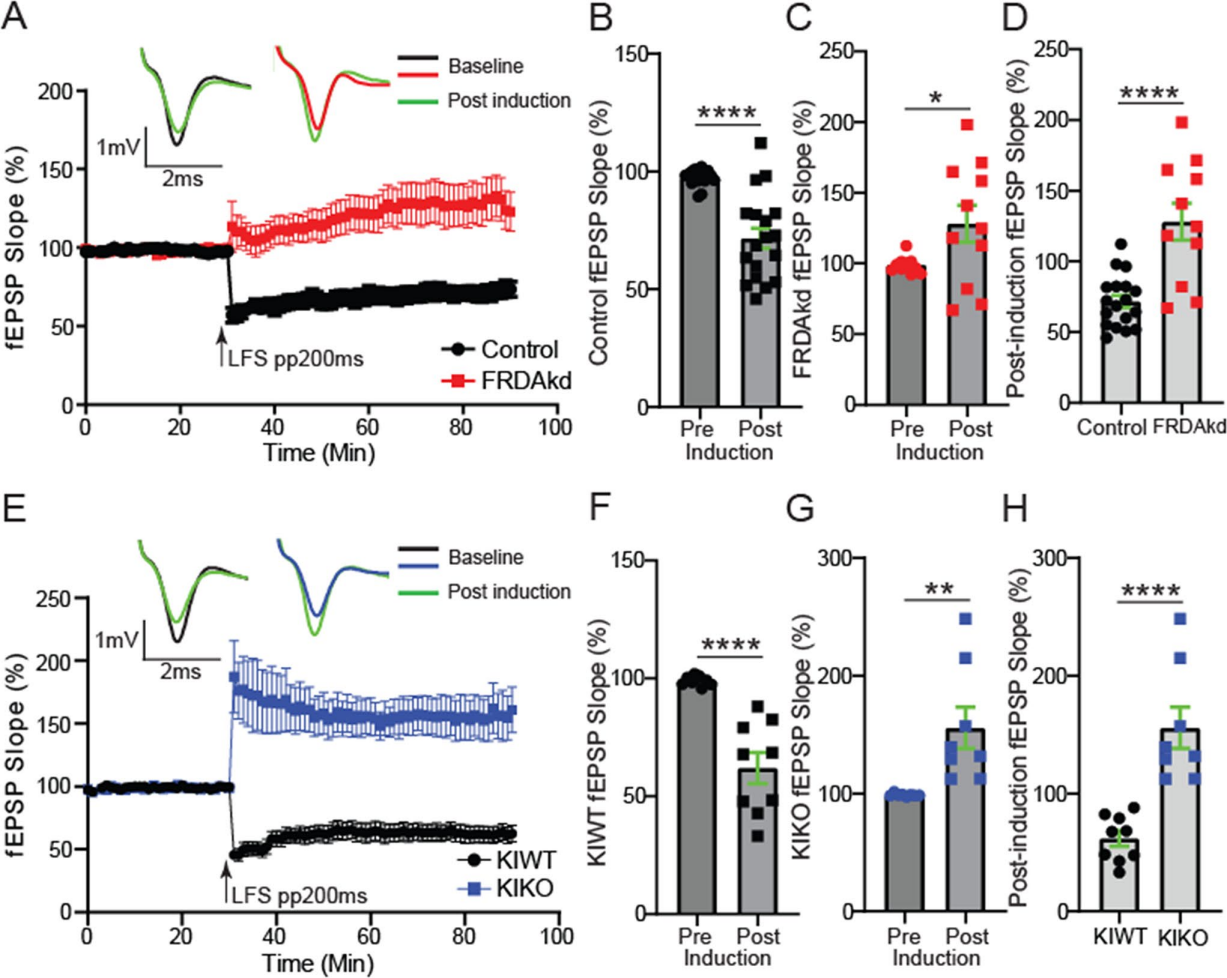
FRDA mice display similar impairments in NMDA-dependent LTD. (**A**) Time course of Parallel fiber-Purkinje cell synapse fEPSP slope 30 min before and 60 min after a train of paired stimuli of 200ms interval delivered at 1 Hz for 15 min in control and FRDAkd mice. Top traces: representative traces of fEPSP recorded at baseline and after LTD induction. (**B**,** C**) Histograms of percent changes in fEPSP following LTD induction in control (**B**) and FRDAkd (**C**) mice relative to baseline. (**D**) Comparative analysis of percent change in post-induction fEPSP magnitude between control and FRDAkd mice. (**E**) Time course of Parallel fiber-Purkinje cell synapse fEPSP slope 30 min before and 60 min after the train of low frequency 200ms paired stimulation protocol in KIWT and KIKO mice. Top traces: representative traces of fEPSP recorded at baseline and after LTD induction. (**F**,** G**) Histograms of percent changes in fEPSP following LTD induction in KIWT (**F**) and KIKO (**G**) mice relative to baseline. (**H**) Comparative analysis of percent change in post-induction fEPSP magnitude between KIWT and KIKO mice. The last 10 min of baseline and/or post-induction timelines were used for all comparative analyses. *P < 0.05, **P < 0.01, ****P < 0.0001; Control, FRDAkd: *n* = 18/18, 11/11, slices/mice; KIWT, KIKO: *n* = 9/9, 8/8, slices/mice, slices/mice. Data are given as mean ± SEM and analyzed by analyzed by the 2-tailed unpaired Student’s t test

In assessing an NMDA-receptor-based form of LTD [24], using the same baseline to post-induction comparative approach as above, PP200ms-LFS reliably induced LTD in uninduced FRDAkd mice (Fig. 8A-B; -25.9 ± 4.4%; *P* < 0.0001, *n* = 18/18, slices/mice). As observed for the PP50ms-LFS protocol, induced FRDAkd mice exhibited potentiation of the fEPSP slope magnitude rather than depression (Fig. 8C and 29.3 ± 13.1; *P =* 0.037, *n* = 11/11, slices/mice). The post-tetanic responses of the last 10 min showed a pronounced potentiation in the induced mice (Fig. 8D and 56.5 ± 11.5; *P* < 0.0001, Control, FRDAKD: *n* = 18/18, 11/11 slices/mice). As expected, application of the PP200ms-LFS protocol on slices from KIWT mice induced a pronounced depression of the fEPSP slope response (Fig. 8E-F; -37.1 ± 6.55; *P* < 0.0001, *n* = 9/9, slices/mice). Like in the induced FRDAkd mice, stimulation of PF with this protocol resulted in potentiation of the fEPSP slope magnitude instead of depression (Fig. 8G and 56.9 ± 17.6; *P =* 0.0060, *n* = 8/8, slices/mice). Finally, we compared the last 10 min of post-tetanus fEPSP slope magnitude between the genotypes and found a significant potentiation in the KIKO mice (Fig. 8H and 93.9 ± 17.9%; KIWT, KIKO: *n* = 9/9, 8/8, slices/mice; *P* < 0.0001). Altogether, loss of FXN in cerebellar circuits altered long-term plasticity, with LTP and LTD protocols in both FRDA models unexpectedly resulting in the opposite direction of plasticity compared to controls.

## Discussion

In the present study, field recording of synaptic responses in two FRDA mouse models reveals concordant impairments in basal synaptic transmission and long-term synaptic plasticity. As there were slightly disparate impacts on mitochondrial markers and PC survival in the two models, these results indicate that cerebellar synaptic function is significantly impacted independent of common structural or mitochondrial alterations. The neurological symptoms of FRDA are historically associated with early degeneration of DRG and later loss of deep cerebellar nuclei (In neurons), but recent fMRI studies demonstrate functional defects in cerebellar regions with minimal structural damage and histologic evaluations suggest some degree of PC abnormalities [14, 15, 17, 18]. Thus, human studies support investigation into the physiological importance of PC dysfunction in FRDA.

Consistent with our previous findings, FXN levels are prominent in PC, a finding conserved across species and given the importance of frataxin in ATP production, this finding is expected. Loss of FXN should, therefore, lead to PC-mediated cerebellar dysfunction. In the present experiments, the FRDAkd (severe, subacute FXN loss) and the KIKO (congenital, modest FXN loss), models change differently in the levels of mitochondrial proteins compared to their respective untreated and KIWT controls, which showed normal FXN expression and basic synaptic transmission parameters relative to regular C57BL/6 mice in our pilot studies. Both models display lower levels of FXN as expected and unaffected levels of ATP5A but have variable effects on other mitochondrial markers. As immunological assays do not capture enzyme activity, these results provide an incomplete assessment of mitochondrial function. Still, the differences between models and the modest magnitude of changes suggest that specific mitochondrial abnormalities alone do not drive the concordant synaptic defects. Similarly, while the inducible FRDAkd mice exhibit a 25% reduction in PC density, these cells are preserved in KIKO mice, suggesting that PC degeneration is either a late effect in FRDA or found only at the extreme of the phenotype. In agreement with this notion, the density of PC in the cerebellar cortex of FRDA patients is well preserved, albeit with signs of minor structural injuries, again suggesting cell death is not the primary driver of pathology [1, 14, 15].

In extracellular recordings of PC cell responses to PF stimulation, I/O curves demonstrate prominent hypo-excitability in both FRDAkd and KIKO mice, but with slightly different amplitudes which is likely attributable to the differences in the ages of the mice (16–18 months KIKO/KIWT mice and in 8–9 months old FRDAkd/KT mice). Our results are consistent with observations in various spinocerebellar ataxia rodent models [31, 32]. The hypoexcitability in PF-PC synaptic transmission could be due to changes in probability of neurotransmitter release by PFs, but the paired-pulse ratio was indistinguishable in both FRDA mouse models, indicating that the presynaptic release machinery was like unaffected by the loss of FXN. Together, these data suggest that altered PC synaptic activity contributes to the neurological phenotype in FRDA but future work involving whole-cell patch clamp paired recording of PCs and inhibitory interneurons such as basket, stellate, Golgi and DCN interneurons would address the contribution of direct excitatory or inhibitory inputs to hypo-excitability of PF-PC synapses.

LTP and LTD at the PF-PC synapse in the cerebellum are important cellular substrates for motor learning [33, 34, 35, 36, 37]. The mechanisms of these two forms of plasticity are diverse, with the expression of distinct forms of LTP and LTD depending on induction patterns, intensity, and functional implications [38]. We tested several low and moderate frequency protocols that failed to consistently induce PF-PC LTP or LTD. However, a high-frequency (4 × 200 Hz trains of 500ms duration, 5 min intervals) protocol [28] readily induces PF-PC LTP in control mice, whereas LTD is consistently induced by stimulation with 900 paired-pulses (50 or 200ms intervals) delivered at 1 Hz. Interestingly, application of these protocols on cerebellar slices from FRDAkd and KIKO mice switches the direction of the expected plasticity, with the LTP induction protocol producing LTD and the LTD one producing LTP in both FRDA mouse types.

PF-LTD is classically induced by concomitant activation of PF and CF tracts [39]. Activation of the CF tracts is thought to induce complex spikes and large Ca^2+^ influx in PCs [40, 41], where the increase in intracellular Ca^2+^ concentration is necessary for the induction of LTD [42]. This requirement of CF activation can be replaced either by direct depolarization of PCs to levels sufficient to allow calcium influx through voltage-gated calcium channels [43, 44, 45, 46]. Still, activation of PFs using TBS and LFS induction protocols can induce PF-LTD without synergistic activation of these CF tracts [47, 48] provided that PF stimulation is strong enough to mobilize Ca^2+^ within the PC [47]. Thus, our observation that activation of PF with paired-pulse protocols delivered at 1 Hz for 15 min induced LTD in the absence of synergistic activation of CF is consistent with the recent findings on cerebellar LTD. While future studies are needed for comparative analysis of the Ca^2+^ dynamic changes driven by distinct induction protocols, our results suggest that mobilization of Ca^2+^ levels within PCs necessary for LTD might be deficient in FRDA mice.

While long-term plasticity at cerebellar and hippo/cortical synapses appears to rely on similar pathways [36, 49], different protocols could activate different cerebellar pathways. We chose a non-classical LTD protocol that reliably work to assess plasticity as the typical protocols did not work in our older mice. Furthermore, the diversity in plasticity mechanisms makes it difficult to make a generalized conclusion. Therefore, our results should be viewed within the scope of the plasticity induced by our protocols, which may well be specific to the age group and induction patterns used in this study.

The distinct mechanisms underlying long-term plasticity in the cerebellum may differ with the locus of expression, the particular synapses, age, and prior history of synaptic use. To gain some mechanistic insights into the locus of synaptic plasticity defects in FRDA mice, we used the PPF paradigm. Using that approach, applying two pulses at intervals ranging from 10 to 200 to measure the paired-pulse facilitation ratio produces no change at any interval tested following application of LTP and LTD induction protocols, indicating that dysfunction at the postsynaptic end of the PC is likely the origin of the observed synaptic deficits in both FRDA models. Levels of CaMKII are not significantly different in the FRDA mice, albeit both genotypes display trends toward reduced expression, and the present approach has limited sensitivity for detecting synaptic activity changes in CaMKII. These results suggest that LTP and LTD defects in both models share the same loci of synaptic deficits, but do not fully resolve the role of in a CaMKII in such processes.

However, we must emphasize that the long-term plasticity protocols employed in this study represent the initial steps in a sequence of events leading to a change in synaptic efficacy and those events are likely contributors to the circuit abnormalities. The lack of change in CaMKII level of expression in the two FRDA models suggest that any role by this kinase would be driven by phosphorylation activity rather than actual availability. Given that both LTP and LTD can be induced in FRDA mice, albeit with unexpected protocols, disruption in the initial entry of Ca^2+^ through glutamate receptors and VGCC channels [50, 51] upstream of CaMKII may be one of the many players orchestrating these synaptic alterations. However, dysregulation of ion channels localized on the ER and mitochondrial membrane could lead to alterations in Ca^2+^ handling properties of these intracellular stores, leading to a switch in the typical Ca^2+^ threshold generated by these induction protocols. Future studies are needed to investigate the role of these Ca^2+^ pumps, with special focus on the calcium ATPase (PMCA) and Na^+^-Ca^2+^ exchanger pumps, sequestration into intracellular stores via smooth endoplasmic reticulum calcium ATPase (SERCA), mitochondria uniporter (MICU), and Ca^2+^ binding proteins [52]. While intracellular Ca^2+^ is a measurable and fundamental molecule in this dynamic process, an extensive number of signal transduction molecules downstream of CaMKII including protein kinase C (PKC), the cyclic adenosine monophosphate-dependent protein kinase (PKA), (Erk)/mitogen-activated protein kinase (MAPK), Src kinase, nitric oxide (NO), protein phosphatase 2 A, and calpain have been suggested to play a role in translating the Ca^2+^ signal into the long-lasting changes in synaptic strength [53, 54, 55, 56, 57]. Calpain I also plays a role in ataxia in calpain I knockout mice and can act on many or the receptors at cerebellar synapses. Thus, careful evaluation of the involvement of these signal transduction pathways in the switch in the form of plasticity observed here is needed for a full understanding of the molecular mechanisms of the circuit abnormalities in FRDA mice.

The most salient observation in this study is the polarity switch in long-term plasticity in the two FRDA mouse models, reminiscent of the effects of deletion of the CaMKII protein on the polarity of long-term plasticity [58]. However, the lack of significant changes in CaMKII expression in the present study suggest that the noted defects are unrelated to its absolute levels. Still, deficiencies in its activation by Ca^2+^ disrupt long term plasticity. Indeed, the threshold setting properties of CaMKII operate within a narrow Ca^2+^ concentration range [58, 59, 60], with PF-PC LTD preferably induced at local calcium concentrations above this threshold and postsynaptic LTP at much lower concentrations [60, 61, 62, 63]. Notably, intracellular application of a low concentration of the Ca^2+^ chelator BAPTA induces LTP following induction with an LTD protocol, whereas intracellular increases of Ca^2+^ load photolytically turn an LTP-inducing protocol into LTD [49], resembling our observations in FRDA mice. Conceivably, stimulation of PFs alone with our LTP protocol likely induces high intracellular Ca^2+^ levels in PCs that favor LTD in FRDA mice, whereas activation of these fibers alone with our LTD induction protocol likely produces low level of intracellular Ca^2+^ levels that favor LTP in these mice. Alternatively, dysfunction in the activation of signal transduction pathways downstream of intracellular Ca^2+^ could also play a pivotal role in the switch in synaptic polarity reported here. In support of this notion, cerebellar LTD induction has been shown to be dependent on negative regulation of phosphatases by the activation of PKC and CaMKII [65], whereas LTP appears to depend on the positive regulation of these protein phosphatases [66]. Thus, it is conceivable that Ca^2+^ handling deficits in the FRDA mice altered the sensitivity of these opposing signal transduction pathways to intracellular Ca^2+^ levels, leading to a switch in polarity of long-term plasticity normally associated with our induction protocols. Comprehensive Ca^2+^ imaging, biochemical, electrophysiological, and immunohistochemical studies in combination with pharmacology are needed to interrogate the various elements of the complex machinery of long-term plasticity. Such studies would supplement our CaMKII expression studies here and provide significant insights into how PCs in FRDA mice integrate global Ca^2+^ transients and modulate the diverse signal transduction pathways to produce the switch in polarity of plasticity in these mice. Although intracellular Ca^2+^ dysregulation is a common feature in cells from FRDA mice [67], future work involving combined two-photon imaging and whole-cell patch clamp recording of long-term plasticity might elucidate how such induction protocols influence Ca^2+^ dynamics as well as their interaction with kinase and phosphatase pathways.

In the present study, by recording extracellular PF-PC fEPSPs in the molecular layer, we showed concordant net decrease in basal excitatory drive on PCs along with a switch in polarity of long-term plasticity associated with two induction protocols in both FRDA mouse models at the opposite end of the phenotypic spectrum. These findings implicate PF-PC dysregulation in the pathophysiology of FRDA. Future work involving in vivo recordings combined with optogenetic manipulations of cellular activity would address the causal contribution of the synaptic impairments reported here to the motor incoordination in FRDA mice and by analogy in human FRDA.

## Electronic supplementary material

Below is the link to the electronic supplementary material.

Supplementary Material 1

## Data Availability

All data supporting the findings of this study are available within the paper and its Supplementary Information.

## Abbreviations

FRDA: Friedreich’s ataxia
FXN: Frataxin
KIKO: Knock in-knockout
FRDAkd: Friedreich’s ataxia knockdown
PC: Purkinje cell
Fe/S: Iron-sulfur clusters
fMRI: Functional magnetic resonance imaging
PBS: Phosphate buffered saline
TBS-T: Tween/Tris-buffered saline
PF: Parallel fiber
I-O: Input-output
GRP75: 75-kDA glucose-regulated protein
TFAM: Mitochondrial transcription factor A
TOM20: Translocase of Outer Mitochondrial Membrane 20
PPR: Paired-pulse ratio
HFS: High frequency stimulation
PP: Paired-pulse
LFS: Low frequency stimulation
LTP: Long-term potentiation
LTD: Long-term depression
CaMKII: Calcium–calmodulin (CaM)-dependent protein kinase II
DAPI: 4’,6-diamidino-2-phenylindole
PSD93: Postsynaptic Density Protein 93
CB: Calbindin D28K
BCA: Bicinchoninic acid
SDS: Sodium dodecyl sulfate
PBS: Phosphate buffered saline
AMPAR: α-amino-3-hydroxy-5-methyl-4-isoxazolepropionic acid receptor
NMDAR: N-methyl-D-aspartate receptor
ANOVA: Analysis of variance
CHOP: Children’s Hospital of Philadelphia

## Declarations

### Generative AI in Scientific Writing

The authors declare that generative artificial intelligence (AI) and AI-assisted technologies were not used in the writing process of this manuscript.
