# Supplementary figures and images for: Functional Characterization of Parallel Fiber-Purkinje Cell Synapses in Two Friedreich’s Ataxia Mouse Models
Source: Cerebellum. 2025 Feb 5;24(2):42. doi: 10.1007/s12311-025-01796-0 (PMC11799031; doi:10.1007/s12311-025-01796-0)

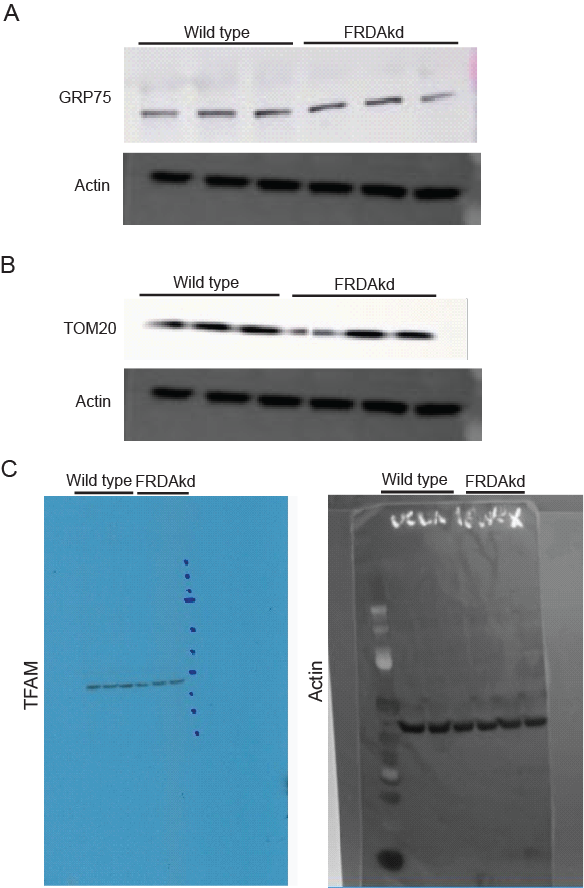


**Supplemental figure**

Supplement: Supplementary file 1 — Supplementary Material 1 [file 12311_2025_1796_MOESM1_ESM.docx]
